# The hepatoprotective effect of 4-phenyltetrahydroquinolines on carbon tetrachloride induced hepatotoxicity in rats through autophagy inhibition

**DOI:** 10.1186/s40659-024-00510-4

**Published:** 2024-05-27

**Authors:** Mohamed Hussein Abdelgalil, Reem H. Elhammamy, Hanan M. Ragab, Eman Sheta, Ahmed Wahid

**Affiliations:** 1 Department of Pharmaceutical Biochemistry, Faculty of Pharmacy, Alexandria University, Alexandria, Egypt; 2 Department of Pharmaceutical Chemistry, Faculty of Pharmacy, Alexandria University, Alexandria, Egypt; 3 Department of Pathology, Faculty of Medicine, Alexandria University, Alexandria, Egypt

**Keywords:** 4-phenyltetrahydroquinoline, Tacrine derivatives, Liver injury, CYP2E1, Autophagy, HepG2, Hepatoprotective, CCL_4_-induced hepatotoxicity

## Abstract

**Background:**

The liver serves as a metabolic hub within the human body, playing a crucial role in various essential functions, such as detoxification, nutrient metabolism, and hormone regulation. Therefore, protecting the liver against endogenous and exogenous insults has become a primary focus in medical research. Consequently, the potential hepatoprotective properties of multiple 4-phenyltetrahydroquinolines inspired us to thoroughly study the influence of four specially designed and synthesized derivatives on carbon tetrachloride (CCl4)-induced liver injury in rats.

**Methods and results:**

Seventy-seven Wistar albino male rats weighing 140 ± 18 g were divided into eleven groups to investigate both the toxicity profile and the hepatoprotective potential of 4-phenyltetrahydroquinolines. An in-vivo hepatotoxicity model was conducted using CCl4 (1 ml/kg body weight, a 1:1 v/v mixture with corn oil, i.p.) every 72 h for 14 days. The concurrent treatment of rats with our newly synthesized compounds (each at a dose of 25 mg/kg body weight, suspended in 0.5% CMC, p.o.) every 24 h effectively lowered transaminases, preserved liver tissue integrity, and mitigated oxidative stress and inflammation. Moreover, the histopathological examination of liver tissues revealed a significant reduction in liver fibrosis, which was further supported by the immunohistochemical analysis of α-SMA. Additionally, the expression of the apoptotic genes BAX and BCL2 was monitored using real-time PCR, which showed a significant decrease in liver apoptosis. Further investigations unveiled the ability of the compounds to significantly decrease the expression of autophagy-related proteins, Beclin-1 and LC3B, consequently inhibiting autophagy. Finally, our computer-assisted simulation dockingonfirmed the obtained experimental activities.

**Conclusion:**

Our findings suggest that derivatives of 4-phenyltetrahydroquinoline demonstrate hepatoprotective properties in CCl4-induced liver damage and fibrosis in rats. The potential mechanism of action may be due to the inhibition of autophagy in liver cells.

## Background

The liver is a vital organ that plays a fundamental role in supporting physiological processes through various metabolic activities [1]. It actively participates in the metabolism and processing of drugs and other foreign substances, utilizing its metabolic capabilities to break them down and facilitate their excretion from the body [2]. Cytochrome P450s (CYPs) constitute a superfamily of enzymes involved in the bioconversion of a wide range of endogenous and exogenous compounds in the liver [3]. Among these numerous CYPs, cytochrome P450 2E1 (CYP2E1) holds a unique position in liver pathophysiology [4, 5].

Liver fibrosis is a pathological condition characterized by the abnormal accumulation of extracellular matrix proteins (ECM) in the liver due to chronic injury or inflammation [6]. During liver injury, hepatic stellate cells (HSCs) undergo phenotypic changes and are transformed into myofibroblasts, which participate in the excessive production of ECM [7]. Autophagy is another important mechanism for maintaining homeostasis by breaking down and recycling damaged organelles and other cellular components [8]. This process is mediated by the activation of autophagy-related proteins, such as Beclin-1 and microtubule-associated protein 1 light chain 3 (LC3B) [9]. Lipophagy is a specialized form of autophagy that regulates lipid metabolism by selectively engulfing and degrading lipid droplets within cells. It is involved in the activation of HSCs and the progression of fibrosis as it supplies them with the energy needed for their activation [10, 11]. In this context, autophagy inhibition has emerged as a promising targeted pathway for the development of hepatoprotective agents [9–11].

Carbon tetrachloride (CCl_4_) is a prevalent toxic chemical that can induce immediate damage to the liver [12]. The enzymatic action of CYP2E1 initiates the metabolism of CCl_4_ within the liver. This enzymatic reaction leads to the formation of highly reactive radicals such as trichloromethyl (CCl3⋅), which triggers an entangled interaction of oxidative stress, inflammation, apoptosis, and fibrosis, making CCl_4_-induced hepatotoxicity a widely used model for evaluating hepatoprotective agents [13, 14].

**Fig. 1:**
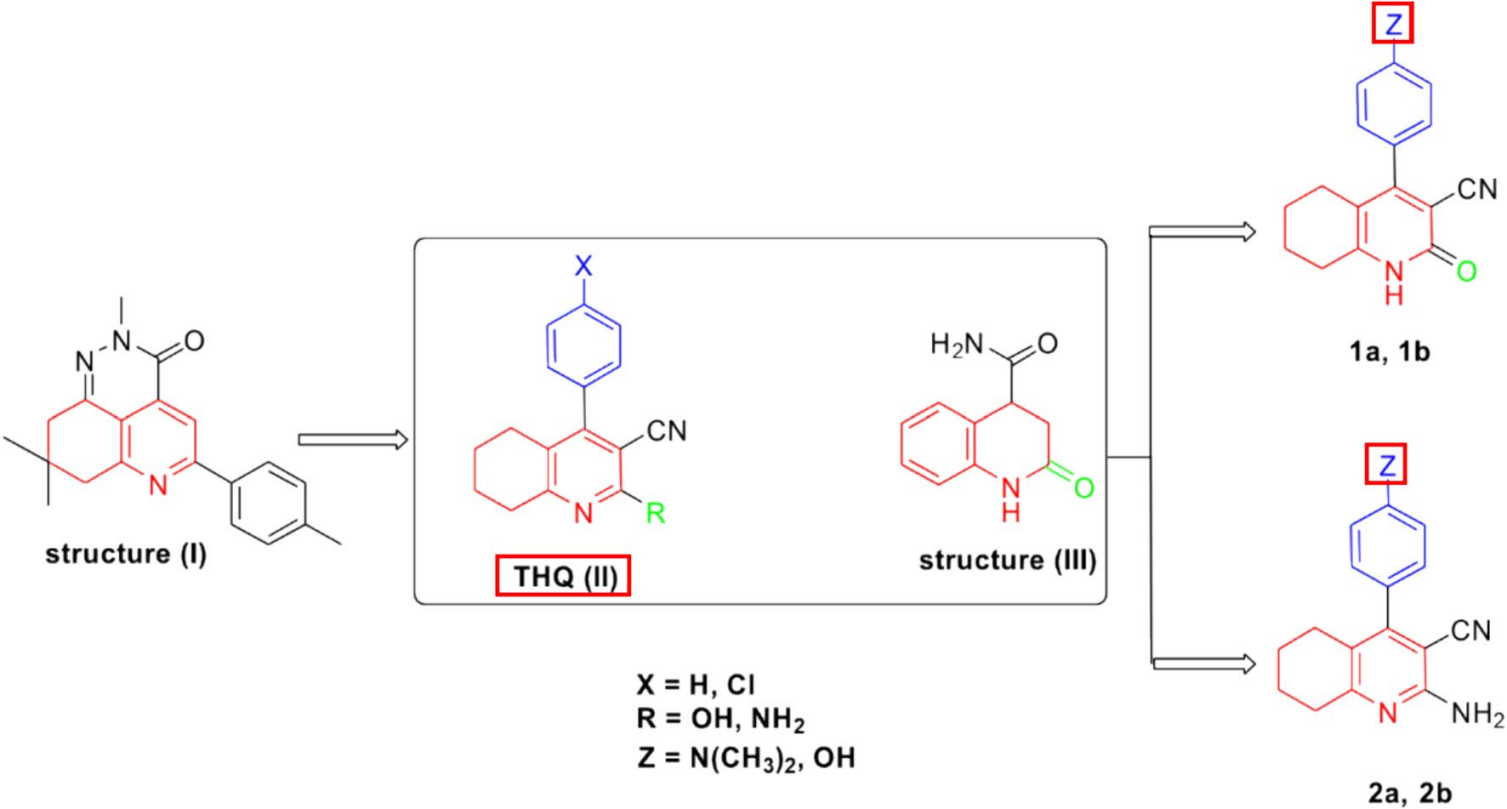
Rational for the synthesis of compounds **1a, 1b, 2a**, and **2b**

Tetrahydroquinoline (THQ) derivatives hold a considerable significance in medicinal chemistry. Owing to their diverse pharmacological activities, THQs are often used as scaffolds for designing several bioactive compounds, encompassing antimicrobial, anticancer, anti-inflammatory, and acetylcholinesterase inhibitory activities [15–19]. Tetrahydroquinolines have been also reported as an important scaffold possessing hepatoprotective properties (**Structure I**, Fig. 1) [20]. In addition, the discovery of the ability of several 4-phenyltetrahydroquinoline derivatives (**THQ II**, Fig. 1) synthesized in our laboratories [18, 21, 22] to decrease elevated levels of ALT in tested rats further drew our attention to the importance of such a moiety. The structure of these compounds was inspired by the acetyl cholinesterase inhibitor (AChEI), tacrine. Finally, reviewing the literature further emphasized the importance of tetrahydroquinoline derivatives as hepatoprotective agents (**Structure III**, Fig. 1) [23, 24].

Studying the structures of the above-mentioned compounds revealed the importance of the presence of substituents at the 2- and 4-positions of the tetrahydroquinoline nucleus. Therefore, all the aforementioned discoveries have motivated us to design and synthesize four compounds (**1a, 1b, 2a**, and **2b**) bearing different substituents on the phenyl group present in our previously designed 4-phenyltetrahydroquinolines in addition to different substituents at the 2-position of the pyridine ring for further investigation of the effect of these substitutions on the hepatoprotective activities of the synthesized compounds. Consequently, the objective of this study is to explore the hepatoprotective effect of these four novel 4-phenyltetrahydroquinoline derivatives and assess their safety profile on hepatocytes. Moreover, we aim to elucidate the potential mechanism of action underlying their effect and their structure–activity relationship.

## Materials and methods

### Chemicals and drugs

Dimethyl sulfoxide (DMSO) was purchased from Thermo-Fisher Scientific (USA). Both Dulbecco’s Modified Eagle Medium (DMEM) and fetal bovine serum (FBS) were obtained from Biowest (France). Tacrine was procured from Sigma-Aldrich Co. (St. Louis, MO, USA). Corn oil and carbon tetra chloride (CCl_4_) were both obtained from Alpha Chemika (India). Carboxymethylcellulose sodium salt (CMC) as well as formalin were purchased from Adwic—El Nasr Pharmaceutical Co. (Egypt). Silymarin and isoflurane 100% were both acquired from Pharco-pharmaceuticals Inc. (Egypt). Phosphate buffered saline was obtained from Loba chemie (Mumbai, India).

### Compounds synthesis

All reagents and solvents were purchased from commercial and international suppliers. Compound melting points in open glass capillaries were obtained using the Thomas–Hoover melting point equipment. Thin-layer chromatography (TLC) using silica gel-precoated aluminum sheets (Type 60 GF254; Merck; Germany) were used to monitor the reactions and determine the purity of the chemicals employed in the study; compounds were identified by exposing spots on TLC sheets to an ultra-violet lamp at k = 254 nm for a few seconds.

**Preparation of 4-aryl-1,2,5,6,7,8-hexahydro-2-oxoquinoline-3-carbonitrile (1a and 1b)**

Equimolar amounts of the appropriate aromatic aldehyde (4.5 mmol), 4.41 g cyclohexanone (4.5 mmol, 0.466 mL), 0.508 g ethyl cyanoacetate (4.5 mmol, 0.478 mL), and 0.693 g ammonium acetate (9 mmol) were heated under reflux in ethanol (10 mL) for 5 h. Most of the solvent was evaporated to give a concentrated solution, which was allowed to stand for 24 h. The desired compound precipitated out as colored crystals, which were filtered, washed with ethanol, dried, and recrystallized in ethanol (18).

**4-(4-(dimethylamino)phenyl)-1,2,5,6,7,8-hexahydro-2-oxoquinoline-3-carbonitrile (1a)**

Yellow red crystals; yield 45%; melting point (m.p.): 233–235 °C, ^1^H NMR (300 MHz, CDCl_3_): δ 1.66–1.81 (m, 4H, C_6_-H_2_ and C_7_-H_2_), 2.38–2.39 (dist. t, 2H, C_5_-H_2_), 2.57 (s, 6H, N(CH_3_)_2_), 2.77–2.79 (dist. t, 2H, C_8_-H_2_), 7.47–7.61 (m, 4H, Ar–H) and 11.21 (s, 1H, NH, D_2_O-exchangeable); ^13^C NMR (75 MHz, CDCl_3_): δ 22, 26, 32, 33 (cyclohexyl-C), 57 (N(CH_3_)_2_), 115, 125, 128, 128, 129, 129, 133, 135, 139, 140, 154 and 156 (Ar–C and CN); IR (KBr, cm^−1^): 3137 (NH), 2250 (CN), 1695 (CO); Anal. calcd. For C_18_H_19_N_3_O (293.36): C, 73.69; H, 6.53; N, 14.32. Found: C, 73.58; H, 6.44; N, 14.15.

**1,2,5,6,7,8-hexahydro-4-(4-hydroxyphenyl)-2-oxoquinoline-3-carbonitrile (1b)**

Brownish red crystals; yield 35%; melting point (m.p.): 138–140 °C as reported [25].

**Preparation of 2-amino-4-aryl-5,6,7,8-tetrahydroquinoline-3-carbonitriles (2a and 2b)**

Equimolar amounts of the suitable aromatic aldehyde (4.5 mmol), 0.441 g cyclohexanone (4.5 mmol), 0.297 g malononitrile (4.5 mmol), and 0.693 g ammonium acetate (9 mmol) were heated in dry benzene (25 mL) with stirring under reflux using a Dean-Stark head for 5 h. After removing the solvent, the residue was heated in 5 mL of ethanol. The yellow crystals were separated, washed with ethanol, dried, and recrystallized in ethanol [18].

**2-Amino-4-(4-(dimethylamino)phenyl)-5,6,7,8-tetrahydroquinoline-3-carbonitrile (2a)**

Yellowish-red crystals yield 24%; m.p.: 245–247 °C, as reported [26].

**2-Amino-5,6,7,8-tetrahydro-4-(4-hydroxyphenyl)quinoline-3-carbonitrile (2b)**

Yellowish-red crystals yield 33%; m.p.: 153–155 °C, as reported [25].

## Biochemical and molecular investigations

### Cell culture and MTT assay

Human hepatoma (HepG2) cells were used to evaluate the in-vitro cytotoxicity of the newly synthesized compounds compared to tacrine as their structure-inspiring drug. The HepG2 cell line was obtained from ATCC. The cells were cultured in DMEM with high glucose, supplemented with 10% FBS, 100 U/ml penicillin, and 1% streptomycin were maintained in a 37 °C incubator with 5% CO_2_ in a humidified atmosphere. One day before the treatment, cells were seeded at a density of 1 × 10^4^ cells/well in a sterile flat bottom 96-well tissue culture plate. After leaving the cells to adhere for 24 h, cells were treated with different concentrations (7.8125, 15.625, 31.25, 62.5, 125, 250, and 500 μM) of each compound as well as tacrine for 24 h in the 5% CO_2_ incubator.

Thereafter, cell viability was assessed using a 3-(4,5-dimethyl-2-thiazolyl)-2,5-diphenyl-tetrazolium bromide (MTT) assay obtained from Biobasic inc (Canada) [27]. Following the 24-h treatment period, MTT was added to the wells at a final concentration of 0.5 mg/mL, and the cells were incubated for 4 h in the dark. Subsequently, the media were carefully removed from the wells, and the formed formazan crystals were dissolved by adding 100 μL of DMSO. Absorbance readings were taken using a microplate reader (BioTek, USA), and the percentage cell viability was calculated for each condition relative to the control cells treated with the same DMSO concentration.

### Animal studies

Seventy-seven Wistar albino male rats weighing 140 ± 18 g were obtained from the animal house at the Institute of Graduate Studies and Research, Alexandria University, Egypt. Rats were kept at room temperature, under a 12-h light/dark cycle, in well-ventilated polypropylene cages, and had unrestricted access to food and water. This study was conducted following the ARRIVE guidelines and has been approved (Approval Code: 06-2023-2–26-1-144) by the Animal Care and Use Committee of the Faculty of Pharmacy, Alexandria University.

### Experimental design

Rats were divided into eleven groups, with seven rats in each group. Group I, the control group, received corn oil (1 ml/kg body weight, i.p.) every 72 h for 14 days. Group II, the hepatotoxicity group, received CCl_4_ (1 ml/kg body weight, a 1:1 v/v mixture of CCl_4_ and corn oil, i.p.) every 72 h for 14 days [28]. Groups III–VI, the treated groups, received compounds (**1a, 1b, 2a**, and **2b**), respectively, (25 mg/kg body weight, suspended in 0.5% CMC, p.o.) every 24 h, as well as CCl_4_ (1 ml/kg body weight, a 1:1 v/v mixture of CCl_4_ and corn oil, i.p.) every 72 h for 14 days. Group VII, the positive control group, received silymarin (100 mg/kg, suspended in 0.5% CMC, p.o.) every 24 h, as well as CCl_4_ (1 ml/kg body weight, a 1:1 v/v mixture of CCl_4_ and corn oil, i.p.) every 72 h for 14 days [29]. Groups VIII–XI, the safety profile groups, received only compounds (**1a, 1b, 2a**, and **2b**), respectively, (25 mg/kg, suspended in 0.5% CMC, p.o.) every 24 h for 14 days.

The dose of the newly synthesized compounds (25 mg/kg/day, p.o.) was chosen based on the therapeutic dose of tacrine that maintained the cholinergic-mediated behaviors of rats in a previous study by Goh et al., which investigated the pharmacokinetic and pharmacodynamic properties of tacrine and other cholinesterase inhibitors [30].

### Sample collection and preparation

Rats were euthanized 24 h after the last dose of treatment with an inhaled overdose of isoflurane [31]. Blood was extracted through a cardiac puncture, left for coagulation, and the serum was obtained by centrifugation for 10 min at 5000 rpm (GBF501, Centrifuge Cencom-II, Selecta). Livers were collected, then dissected into portions. One portion was fixed overnight in 10% buffered formalin (Adwic—El Nasr Pharmaceutical Co., Egypt) for histological examination. Another portion was homogenized, centrifuged at 10,000 rpm for 10 min, and the supernatants were collected for further investigation. Total protein content of all homogenates were determined using the Pierce™ BCA Protein Assay Kit (Catalog number: 23227, Thermo-Fisher Scientific Co., USA) following the manufacturer’s instructions [32]. Liver tissues and homogenates were stored at − 80 °C until used.

### Biochemical analysis

A variety of colorimetric serum diagnostic kits were used to assess the toxicity and hepatoprotective potential of the newly synthesized derivatives. Lipid profile parameters, including total cholesterol (TC) and triglycerides (TG), as well as kidney function tests, urea, and creatinine were measured to evaluate the toxicity profile. Liver function tests, including alanine transaminase (ALT), aspartate transaminase (AST), alkaline phosphatase (ALP), and total bilirubin (TBIL) were used for the assessment of both hepatotoxicity and hepatoprotective potential. All diagnostic kits were purchased from Biodiagnostics, Egypt, and were used and stored according to the manufacturer’s instructions.

The level of reduced glutathione (GSH) was measured in liver tissue homogenates using a colorimetric kit for GSH (Cat.No.E-BC-K030-M, Elabscience, USA). Malondialdehyde (MDA) levels were determined as thiobarbituric acid reactive substances (TBARS) in liver homogenates according to the method of Draper and Hadley [33].

### Histopathological assessment

Serial sectioning was done on the formalin-fixed liver tissues, which were then processed into paraffin blocks. Multiple serial, 5-μm-thick sections were cut and mounted on glass slides. One section was stained by H&E stain to assess the activity, while the other was stained by Masson trichrome stain to assess fibrosis. Both activity and fibrosis were assessed using a light microscope (Olympus, CX 22LED) using the METAVIR scoring system [34]. For activity, the number of inflammatory foci (lobular necrosis) and degree of portal inflammation (piece meal necrosis) were evaluated, and then a final histological activity score was given (A0 = none, A1 = mild, A2 = moderate, and A3 = sever). Meanwhile, fibrosis assessment was done on Masson trichrome stained sections. The fibrosis scored according to a 4-tiered system (F0 = no fibrosis, F1 = mild/moderate, F2 = significant, F3 = advanced fibrosis, and F4 = cirrhosis). Other pathologic features, such as the presence or absence of steatosis and/or apoptotic hepatocytes, were assessed if present.

### Immunohistochemistry analysis

From paraffin-embedded blocks of liver tissues, 4- to 5-μm-thick sections were cut using a semi-automated microtome. Sections were mounted on positively charged slides. They were stained by alpha-smooth muscle actin (α-SMA) primary antibody (Clone 1A4, ready-to-use, mouse monoclonal antibody, Dako) using the autostainer DAKO link 48. Stained sections were examined under light microscopy. Five random high-power fields were photographed using a microscope adopted camera. Using Image J software, activated HSCs/myofibroblasts were counted in the five high power fields. HSCs were seen as elongated, flat cells within fibrous septa or liver lobules. Only cells with well-visible nuclei were counted [35].

### Enzyme-linked immunosorbent assay

The pro-inflammatory and pro-fibrotic cytokines tumor necrosis factor alpha (TNF-α) and transforming growth factor beta (TGF-β) were evaluated in liver tissues using ELISA kits (Catalog Numbers: CSB-E11987r and CSB-E04727r, CUSABIO, USA). For further investigation of the potential mechanism of action of the synthesized compounds, the levels of autophagy-related proteins, Beclin-1 (Catalog Number: CSB-EL002658RA, CUSABIO, USA) and LC3B (Catalog No. LS-F19802, Lifespan Biosciences, USA), were quantified in liver homogenates. Furthermore, the activity of the CYP2E1 enzyme was determined in liver cells using an ELISA kit (Catalog Number: CSB-E09782r, CUSABIO, USA).

### Quantitative real-time polymerase chain reaction

Total RNA was extracted from liver tissues using the phenol/guanidine extraction method (miRNeasy Micro Kit, catalog number: 217004, QIAGEN, USA). The cDNA was reverse transcribed from RNA using the Applied Biosystems™ High-Capacity cDNA Reverse Transcription Kit (Catalog Number: 4368814, Thermo-Fisher Scientific). Quantitative real-time PCR was performed to measure the expression of the apoptotic markers BAX and BCL2 using Maxima SYBR Green/ROX qPCR Master Mix 2X (Catalog number: K0221, Thermo-Fisher Scientific) and primer sequences as listed in Table 1. The relative expression of the target gene was calculated according to the threshold cycle (Ct) based on the 2^−∆∆ct^ formula [36].

**Table 1:** Primer sequences used for quantitative real-time PCR

Gene	Forward	Reverse
BCL2	TCGCGACTTTGCAGAGATGT	CAATCCTCCCCCAGTTCACC
BAX	CGTCTGCGGGGAGTCAC	AGCCATCCTCTCTGCTCGAT
β-Actin (house keeping gene)	ATGTGGCTGAGGACTTTGATT	ATCTATGCCGTGGATACTTGG

### In-silico studies and docking protocol

In-silico docking experiments of the synthesized compounds against Beclin-1, LC3B, and CYP2E1 were performed and compared to the observed biological testing results. Molecular Operating Environment (MOEDock 2015) software (Chemical Computing Group, Montreal, QC) was utilized to run computer-assisted simulation docking experiments using an MMFF94X force field. Crystal structures of Beclin-1, LC3B, and CYP2E1 were obtained from the Protein Data Bank (PDB ID: 6TZC, 5GMV, and 3GPH, respectively) [37–39] and were used in the docking simulations. The ligand molecules were constructed utilizing the builder molecule, and the energy was minimized. Ligands were docked within the active site using the MOE Dock.

### Statistical analysis

GraphPad Prism software (version 8.0.2) was used for statistical analysis. For parametric data, results were presented as means ± SEM, and the differences between groups were analyzed using one-way ANOVA followed by Dunnett’s post-hoc test. Histopathological non-parametric data were analyzed using the Kruskal–Wallis test, followed by Dunn’s multiple comparisons test, and presented as medians (minimum to maximum). A p-value < 0.05 was considered statistically significant. The sample size for this study was determined using G*power 3.1 software [40], taking into consideration the different mean values obtained from previous studies in the literature [24, 41, 42]. A power analysis was conducted to determine the minimum sample size required to detect a statistically significant effect with effect size 0.55, α-error 0.05, and power 0.85 [43].

## Results

### In-vitro cytotoxicity assay

**Fig. 2:**
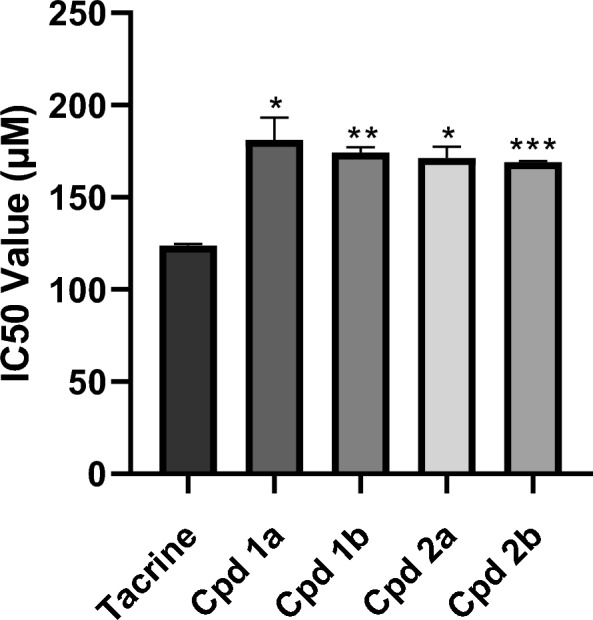
The effect of Cpds (**1a, 1b, 2a**, and **2b**) on HepG2 cells. IC50 values in μM are represented as mean ± SEM. *p < 0.05, **p < 0.01, ***p < 0.001 compared to tacrine

The MTT assay revealed a significant increase in the IC50 values of our compounds (181.15, 174.3, 171.45, and 169.15 μM) for Cpds **1a**, **1b**, **2a**, and **2b**, respectively, compared to tacrine, which had an IC50 of 123.9 μM, as shown in Fig. 2.

### In-vivo toxicity studies

**Fig. 3:**
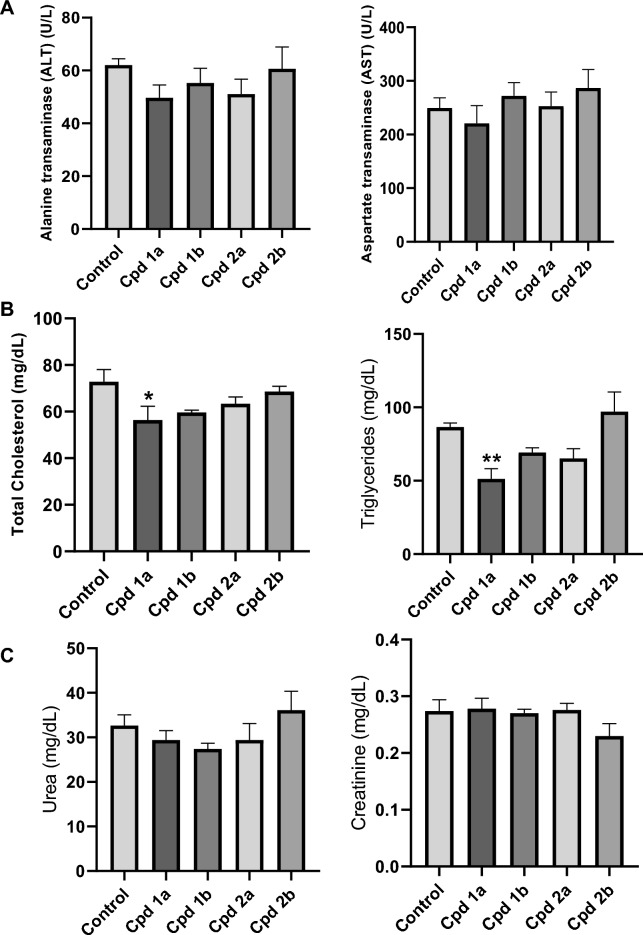
The effect of Cpds (**1a**, **1b**, **2a**, and **2b**) on the Safety Profile. **A** Liver function tests (ALT and AST). **B** Lipid profile (total cholesterol and triglycerides). **C** Kidney function tests (serum urea and creatinine). Values are represented as the mean ± SEM, n = 7. *p < 0.05, **p < 0.01

**Fig. 4:**
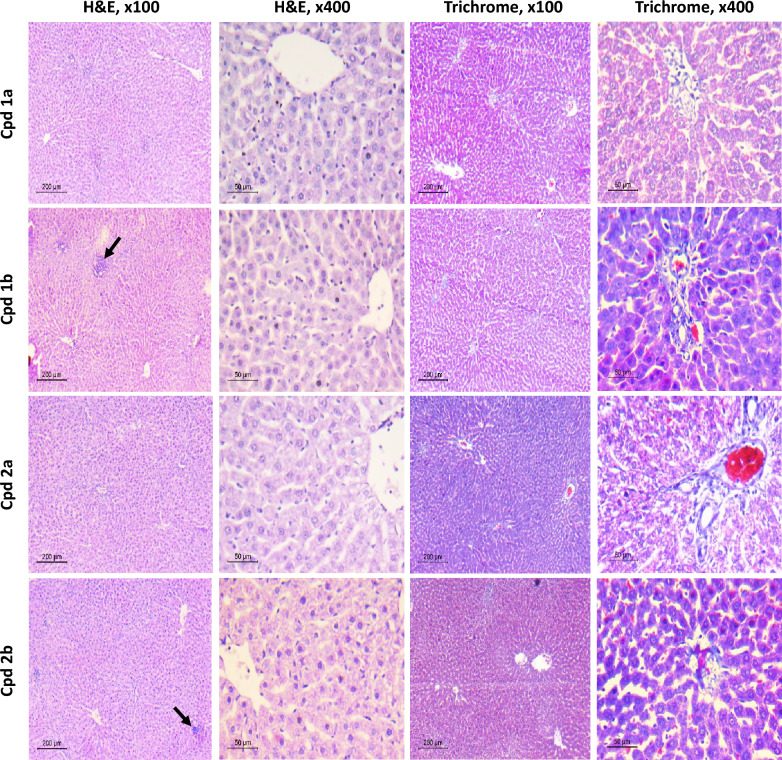
Histopathological assessment of the safety of Cpds (**1a, 1b, 2a**, and **2b**) on liver tissues. H&E stained liver sections (n = 5) show normal liver architecture and normal hepatocytes. Mild portal or lobular inflammation is seen occasionally in Cpd **1b** and **2b** (arrow). Masson trichrome-stained sections (n = 5) show no fibrosis (F0) in low power. High power view of one portal tract shows scanty blue-stained fibrous tissue around portal vessels. Scale bar for low power (× 100) is 200 μM and for high power (× 400) is 50 μM

The oral administration of the tested compounds only, groups (VIII–XI), showed no significant elevation in either of the liver enzymes, ALT or AST, compared to the control group (Fig. 3A). The new compounds also demonstrated no significant increase in total cholesterol or triglyceride levels (Fig. 3B). Nevertheless, Cpd **1a** showed a positive impact on the lipid profile by significantly lowering both total cholesterol and triglyceride levels (Fig. 3B). Moreover, these compounds showed no significant effect on both serum urea and creatinine (Fig. 3C). Furthermore, histopathology assessment of the liver sections of rats showed normal lobular architecture of the liver with no observed fibrosis in any of the liver sections. Meanwhile, mild, insignificant lobular inflammation (activity grade 1) was noted in Cpd **1b** and less frequently in Cpd **2b** (Fig. 4), while no steatosis was noted in any of the treated groups.

### In-vivo hepatoprotective activity

#### 4-phenyltetrahydroquinolines mitigate CCL_4_-induced hepatotoxicity

**Fig. 5:**
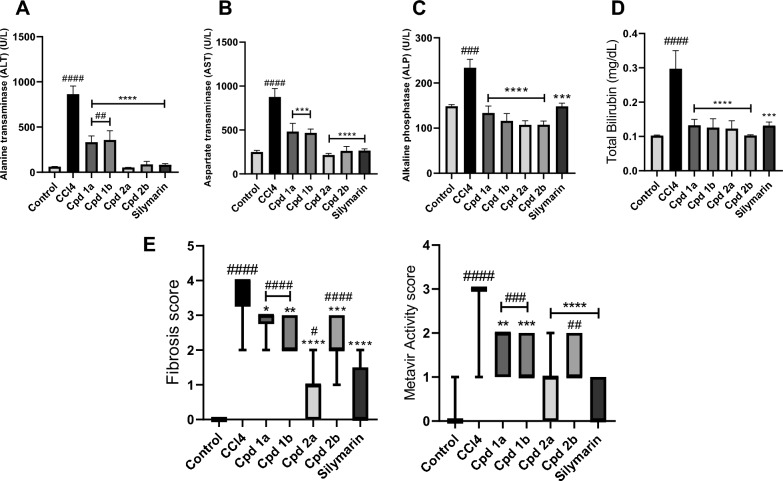
The Effect of Cpds (**1a, 1b, 2a**, and **2b**) on Liver Biomarkers, Degree of Fibrosis and Metavir Activity Scores. **A** Alanine transaminase. **B** Aspartate transaminase. **C** Alkaline phosphatase. **D** total bilirubin. Values are represented as the mean ± SEM, n = 7. **E** The degree of fibrosis and Metavir activity scores in liver tissues using the Metavir scoring system. Results are represented as medians (minimum to maximum), box and whiskers. *p < 0.05, **p < 0.01, ***p < 0.001, ****p < 0.0001 compared to the hepatotoxicity group. ^#^p < 0.05, ^##^p < 0.01, ^###^p < 0.001, ^####^p < 0.0001 compared to the control group

**Fig. 6:**
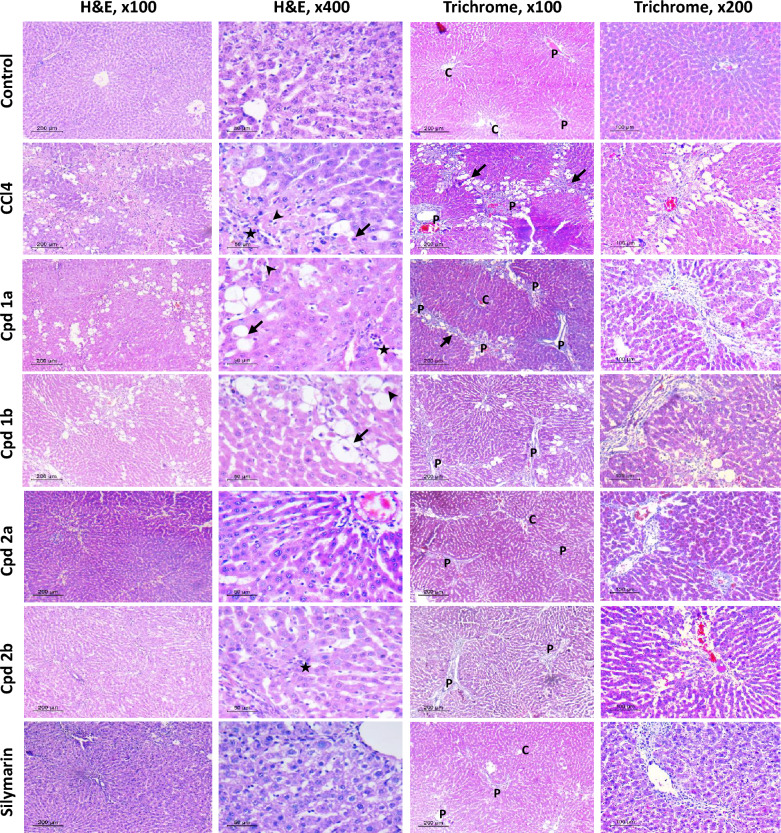
Histopathological assessment of Liver Fibrosis and Metavir activity. Liver sections stained with H&E to assess liver activity, and sections stained with Masson trichrome to assess the degree of fibrosis, n = 7. H&E high power (× 400) shows macrovesicular steatosis (arrows) and apoptotic cells (arrow heads), as well as foci of inflammation (stars). In Masson trichrome stained sections, *P* portal tract, *C* central vein, *arrow* fibrous bridging. Scale bar for low power (× 100) is 200 μM and for high power (× 200) is 100 μM and (× 400) is 50 μM

In the hepatotoxicity group, liver injury was manifested as increased levels of serum ALT, AST, ALP, and total bilirubin (Fig. 5). In the groups treated with the compounds under investigation along with CCL_4_, a significant reduction in ALT, AST, ALP, and total bilirubin levels was observed when compared against the hepatotoxicity group, as shown in Fig. 5. Histological examination of liver tissues was conducted to further validate the aforementioned results using both hematoxylin and eosin (H&E) and Masson’s trichrome stains for accurate assessment of hepatic cell morphology, tissue architecture, and fibrotic changes (Fig. 6). Histopathologic assessment of rats that received corn oil, control group, showed normal histology. The liver showed preserved lobular architecture, where hepatocytes are seen radiating as one- to two-cell-thick cords from central veins. Portal tracts, seen in between lobules, are composed of arteries, veins, and bile ducts within scanty fibrous tissue. No inflammation was noted either in lobules or portal tracts. They were scored as A0 (activity) and F0 (fibrosis) according to the metavir scoring system. Meanwhile, in the hepatotoxicity group, the liver sections showed disorganized architecture with total loss of normal lobular hepatic structure. The liver was formed of multiple nodules of different sizes, separated by thick fibrous septa. The hepatocytes showed evident macrovesicular steatosis in most areas, with scattered apoptotic cells. Lobular necrosis foci were seen, as well as portal and septal moderate inflammation. The liver sections showed a high activity grade (A3) and fibrosis scores (F4). Improvement of both fibrosis and activity scores was noted in different treated groups. Regarding Cpd **1a** treated rats, a mild improvement in the fibrosis score (F3) as well as activity grade (A2) were seen. Macrovesicular steatosis and apoptotic cells were still detected. Meanwhile, Cpd **1b** and Cpd **2b** showed better improvement in fibrosis score. Furthermore, Cpd **2a** showed the best protective effect on the liver sections. The livers are nearing total restoration of hepatic architecture. The fibrosis score was (F0–F1), this was so close to the protective effect of the silymarin group, which dropped the fibrosis score to almost F0. Regarding activity, lobular and portal inflammation improved markedly in Cpd **2a** treated rats. No steatosis or apoptotic cells were detected (Fig. 5E).

#### 4-phenyltetrahydroquinolines alleviate CCL_4_-induced oxidative stress, inflammation, fibrosis, and apoptosis

**Fig. 7:**
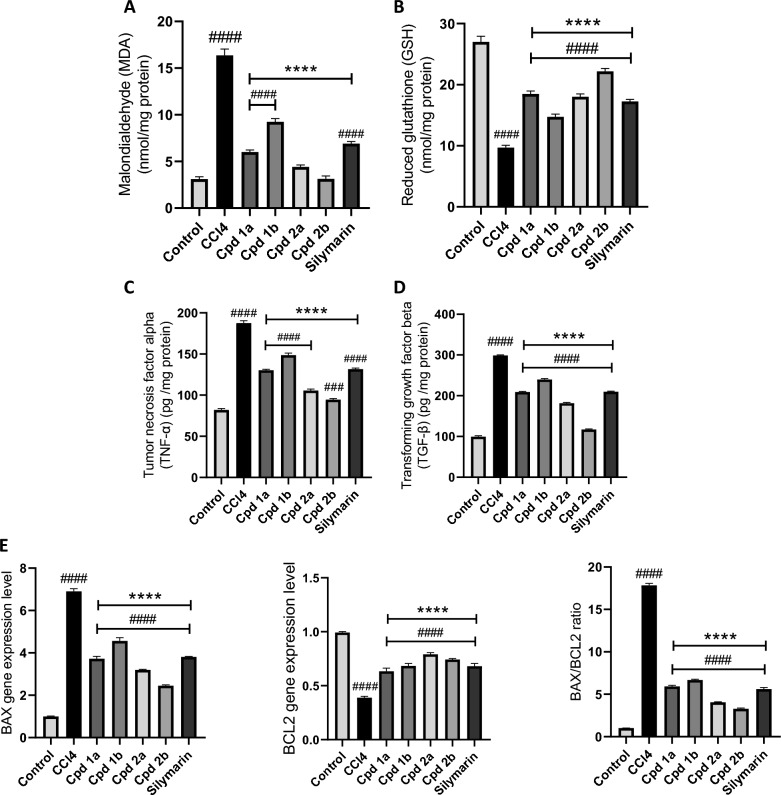
The Effect of Cpds (**1a, 1b, 2a** and **2b**) on oxidative stress, inflammation, fibrosis, and apoptosis. Oxidative stress markers, **A** MDA, and **B** GSH levels were determined spectrophotometrically. The levels of pro-inflammatory, and pro-fibrotic cytokines, **C** TNF-α and **D** TGF-β were quantified using ELISA technique. **E** The level of expression of both the pro-apoptotic and the anti-apoptotic genes, BAX and BCL2, respectively, was determined using qPCR and the ratio of BAX/BCL2 was calculated. Values are represented as mean ± SEM, n = 7. *p < 0.05, ***p < 0.001, ****p < 0.0001 compared to hepatotoxicity group. ^###^p < 0.001, ^####^p < 0.0001 compared to control group

A significant increase in the level of malondialdehyde (MDA) was observed In the hepatotoxicity group, indicating heightened oxidative stress. Concurrently, the treated groups exhibited a significant reduction in MDA levels compared to the hepatotoxicity group (Fig. 7A). Moreover, the hepatotoxicity group displayed a substantial decrease in the level of the endogenous antioxidant, glutathione (GSH), whereas the treated groups maintained elevated GSH levels (Fig. 7B).

**Fig. 8:**
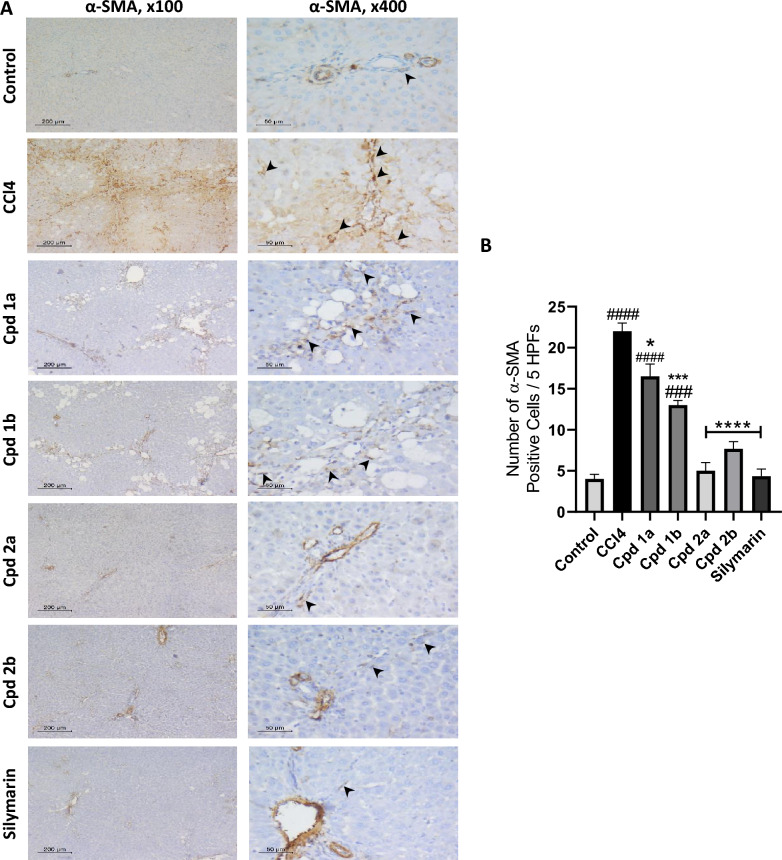
Immunohistochemical assessment of α-SMA in liver tissues. **A** Assessment of α-SMA positive cells in α-SMA immune stained liver section, n = 3. Blood vessels in portal tracts are stained positive as an internal positive control. Arrows are pointing at the positively stained HSCs/myofibroblasts. They are seen as long flat cells in between hepatocytes and within portal tracts. Scale bar for low power (× 100) is 200 μM and for high power (× 400) is 50 μM. **B** Number of α-SMA positive cells / 5 HPFs. Values are represented as mean ± SEM. *p < 0.05, ***p < 0.001, ****p < 0.0001 compared to the hepatotoxicity group. ###p < 0.001, ####p < 0.0001 compared to the control group

The assessment of inflammatory markers, TNF-α and TGF-β, showed a significant increase in both markers within the hepatotoxicity group, while both cytokines exhibited a significant decrease in the treated groups, as demonstrated in Fig. 7C, D, respectively. Additionally, in the immunohistochemical analysis of liver tissues, very few spindle cells were detected in the control group, indicating a low number of α-SMA positive myofibroblasts. In contrast, the hepatotoxicity group exhibited a dark brown stain of fibrotic septa. Upon high-power examination, numerous positive cells were detected within septa and liver lobules, indicating hepatic stellate cell (HSC) activation. This count significantly decreased in the treated groups as shown in Fig. 8.

Quantitative real-time PCR showed an elevated BAX gene expression and decreased BCL2 gene expression in the hepatotoxicity group, resulting in an increased BAX/BCL2 ratio. In contrast, the treated groups demonstrated significantly lower BAX mRNA levels and higher BCL2 mRNA levels, leading to a notable decrease in the BAX/BCL2 ratio (Fig. 7E).

#### 4-phenyltetrahydroquinolines suppress the production of autophagy-related proteins without influencing the activity of CYP2E1

**Fig. 9:**
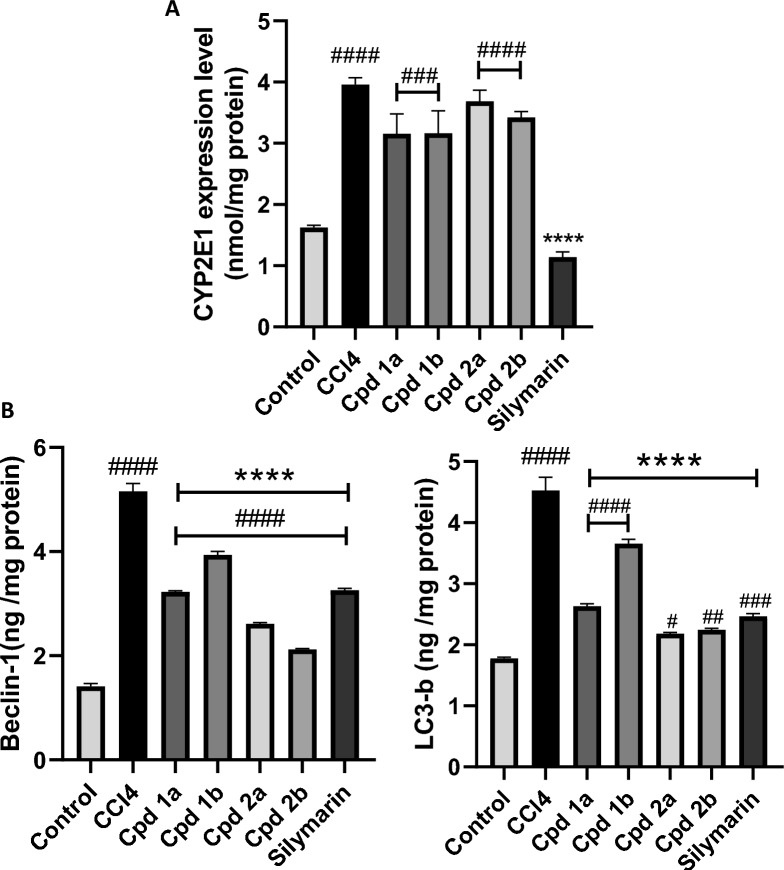
The Effect of Cpds (**1a, 1b, 2a **and **2b**) on CYP2E1 and Autophagy. **A** The levels of CYP2E1 enzyme expression. **B** Autophagy-related proteins, Beclin-1 and LC3B. Protein expression is measured using ELISA technique. Values are represented as mean ± SEM, n = 7. ****p < 0.0001 compared to hepatotoxicity group. ^#^p < 0.05, ^##^p < 0.01, ^###^p < 0.001, ^####^p < 0.0001 compared to control group

According to our findings, as represented in Fig. 9A, our compounds have no significant effect on CYP2E1 enzyme activity. In contrast, silymarin exerts a substantial inhibitory effect on CYP2E1 enzyme activity. Moreover, all four newly synthesized compounds as well as silymarin significantly suppress the expression of both Beclin-1 and LC3B proteins (Fig. 9B).

### In-silico docking studies

**Fig. 10:**
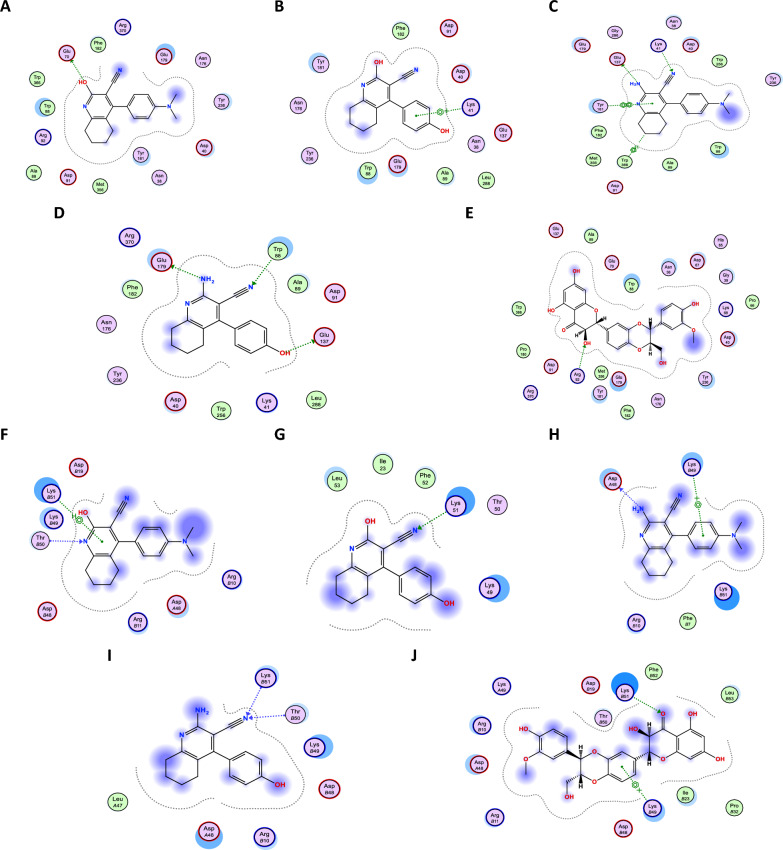
2D Binding mode of compounds **1a, 1b, 2a, 2b** and Silymarin in the binding site of Beclin-1 and LC3B receptors. **A** Compound 1a binding to Beclin-1 receptor. **B** Compound **1b** binding to Beclin-1 receptor. **C** Compound **2a** binding to Beclin-1 receptor. **D** Compound **2b** binding to Beclin-1 receptor. **E** Silymarin binding to Beclin-1 receptor. **F** Compound 1a binding to LC3B receptor. **G** Compound **1b** binding to LC3B receptor. **H** Compound **2a** binding to LC3B receptor. **I** Compound **2b** binding to LC3B receptor. **J** Silymarin binding to LC3B receptor

Docking analysis using crystal structures revealed that compounds **2b** showed the most favorable binding to the Beclin-1 receptor where it formed three hydrogen bonds followed by compound **2a** showing two hydrogen bonds, a pi-H bond and a pi-pi bond with the receptor. On the other hand, compound **1a** and silymarin came third in the binding interactions where they formed one hydrogen bond with Beclin-1. Finally compound **1b** showed only pi-cation interaction with the receptor (Fig. 10A–E). Similarly, the docking simulation of the synthesized compounds with LC3B binding site indicated that all the compounds except for **1b** showed two types of bonds with the binding site: two hydrogen bonds in case of **2b**, one hydrogen bond and one pi-cation bond in case of **2a** and silymarin and a hydrogen bond and a pi–H bond in case of **1a** (Fig. 10F–J).

**Fig. 11:**
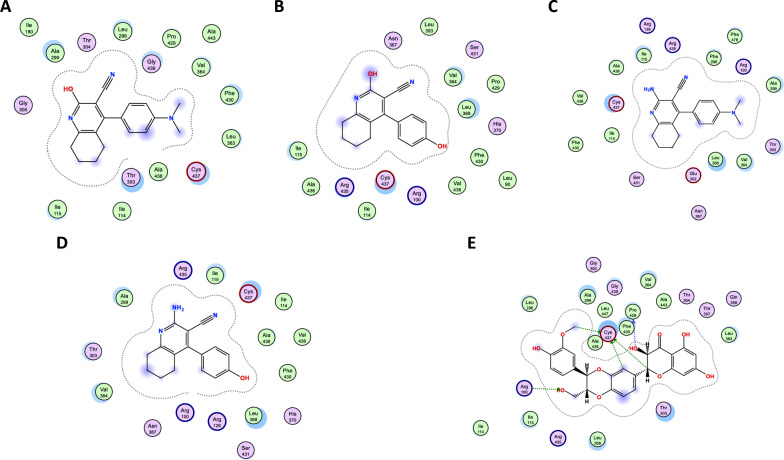
2D Binding mode of compounds **1a, 1b, 2a, 2b** and Silymarin in the binding site of CYP2E1 receptor. **A** Compound **1a**. **B** Compound 1b. **C** Compound **2a**. **D** Compound 2b. **E** Silymarin

Finally, investigating the docking modes of the tested compounds in the CYP2E1 binding sites showed that none of the compounds revealed any bonding with the receptor unlike silymarin which showed five hydrogen bonds with the receptor (Fig. 11).

## Discussion

Liver diseases are widespread throughout the world and have become a significant global health burden [44]. Liver diseases often lead to liver fibrosis, which is an initial histological alteration preceding the progression to cirrhosis that can ultimately lead to hepatocellular carcinoma and death [44, 45]. Carbon tetrachloride (CCl_4_) is a well-known hepatotoxic substance [14]. Owing to the metabolic activity of CYP2E1, CCl_4_ is metabolized into harmful reactive species that cause liver injury through a cascade of complex events involving lipid peroxidation, glutathione depletion, as well as pro-inflammatory and pro-fibrotic cytokines expression upregulation, ultimately leading to cell death and hepatocellular injury [12–14].

Tacrine was the first FDA-approved cholinesterase (ChE) inhibitor for treating Alzheimer’s disease (AD) [46, 47]. However, the lack of sufficient selectivity led to adverse effects, particularly hepatotoxicity [48]. Previous studies demonstrated the hepatoprotective properties of 4-phenyltetrahydroquinolines, which were designed based on the structure of tacrine [18, 21, 22]. The appeal of these compounds stems from their diverse structural and biological characteristics [48–50]. In this study, we investigated the toxicity profile and hepatoprotective effects of four novel 4-phenyltetrahydroquinoline derivatives and their potential mechanisms of action.

HepG2 cells are considered to be a suitable model widely used for studying in-vitro liver toxicity, as they preserve most of the specialized functions of normal human hepatocytes [51]. *In-vitro* cytotoxicity studies of our compounds on the HepG2 cell line revealed the relatively safe hepatic profile of the compounds in comparison to tacrine, showing an almost 1.5-fold increase in the IC50 values regardless of the substituents present on the main scaffold. A significant increase in the IC50 values of the compounds indicate reduced toxicity to liver cells compared to tacrine [52].

A comprehensive assessment of the *in-vivo* toxicity profile of the compounds was conducted, utilizing both biochemical and histopathological analyses for a thorough assessment. Previous studies have suggested that an elevation in transaminases (AST and ALT), bilirubin, or alkaline phosphatase serves as an early indicator of liver damage [53, 54]. Similarly, serum creatinine and urea levels have commonly been employed for the detection of kidney toxicity [55, 56]. Our findings once again demonstrated a safe profile concerning hepatic (ALT and AST) as well as renal (urea and creatinine) biomarkers, with no significant increase observed in these markers levels [18]. The safety profile of the compounds in the liver was further evidenced histologically using hematoxylin and eosin stains, as well as Masson’s trichrome stain, where all compounds showed normal liver architecture and normal hepatocytes. Concerning the lipid profile biomarkers, serum cholesterol and triglycerides, which provided us with insights into potential hepatological issues [57], our synthesized compounds not only proved to be safe in relation to lipid profiles but also some of them exhibited the ability to reduce cholesterol and triglyceride levels. Notably, compound **1a** showed the most favorable results, followed by **2a, 1b**, and, finally, **2b**. As mentioned earlier all compounds could be considered not only safe but also beneficial in some cases regarding the toxicity studies.

As for the hepatoprotective testing, CCl_4_ was used to induce hepatotoxicity in rats. A successful model was obtained as increased levels of liver biomarkers, ALT, AST, total bilirubin, and ALP were observed along with histological changes in liver tissues [28]. Our results indicated that all four compounds were able to significantly reduce the elevated levels of transaminases. Moreover, compounds **2a** and **2b** were as beneficial as silymarin (the reference drug) in reducing the ALT and AST CCl_4_-elevated levels and bringing them back to the control level. Additionally, all compounds showed better results regarding ALP and total bilirubin levels relative to silymarin. For further validation of our biochemical results, a histopathological examination of the liver tissues revealed notable differences among different groups. The control group displayed normal liver histology with intact lobular structure, minimal fibrous tissue, and no signs of inflammation. Conversely, the CCl_4_-treated group exhibited severe liver disorganization, extensive fibrosis, and inflammation [58]. The treated groups showed varying degrees of improvement, with Cpd **2a** demonstrating the most substantial protection, nearly restoring liver normal architecture and significantly reducing inflammation. Importantly, the efficacy of Cpd **2a** closely resembled that of silymarin, which achieved a remarkable reduction in fibrosis and inflammation.

The hepatotoxic effect resulting from CCl_4_ metabolism, mainly triggered by the creation of trichloromethyl radicals, leads to increased level of MDA. The elevated MDA level serves as a clear indicator of intensified lipid peroxidation, and in conjunction with a decrease in the level of the endogenous antioxidant, glutathione, they both provide compelling evidence of oxidative stress in the liver [2, 12]. According to our results, it was evident that compounds **2a** and **2b** were highly superior to compounds **1a**, **1b** and silymarin in reducing MDA levels. In addition, levels of GSH of all tested compounds, regardless of the substituents, were as significantly high as silymarin when compared to the hepatotoxicity group, proving the ability of all compounds to reduce the oxidative stress caused by the reactive oxygen species [59, 60]. Consistent damage to hepatocytes, as a consequence of free radical formation, triggers the recruitment of pro-inflammatory and pro-fibrotic cytokines, ultimately leading to fibrosis [58].

Assessing the pro-inflammatory mediators (TNF-α and TGF-β) revealed that compounds **2a** and **2b** exert a better effect on decreasing the level of both cytokines relative to compounds **1a** and **1b** and silymarin. Persistent liver injury and inflammation stimulate hepatic stellate cell activation, a pivotal process in which quiescent HSCs undergo transformation into myofibroblasts [11]. This activation involves notable changes, including the increased expression of α-SMA and the depletion of lipid droplets within HSCs [9]. α-SMA serves as a marker of HSC activation and plays a central role in the excessive synthesis and deposition of extracellular matrix proteins, thereby contributing to the development of liver fibrosis [61]. In the control group, minimal spindle cells were observed, suggesting a low presence of α-SMA-positive myofibroblasts. In contrast, the CCL_4_ group exhibited intense brown staining in the fibrotic septa. Upon closer examination, a significant number of positive cells were identified within both the septa and liver lobules as an indication of HSCs activation. However, the count of positive cells decreased significantly in the treated groups with compounds **2a** and **2b** showing better levels of significance, closely aligning with silymarin, indicating a decrease in liver fibrosis.

Apoptosis occurs as a consequence of CCl_4_-induced liver damage [14]. The administration of CCl_4_ significantly and markedly upregulated the expression of BAX, an essential pro-apoptotic gene, while concurrently downregulating the expression of the anti-apoptotic gene BCL2. This resulted in an overall elevation of the BAX/BCL2 ratio, indicating an increased apoptotic activity. On the other hand, the treated groups exhibited reduced BAX mRNA levels and increased BCL2 mRNA levels. The notable decrease in the BAX/BCL2 ratio within the treated groups signifies a decrease in apoptosis [13]. Finally, all the results were highly consistent, emphasizing the superiority of compounds **2a** and **2b** to all other compounds, indicating the probable benefits of the presence of an amino group rather than a hydroxy group at the 2-position of the tetrahydroquinoline moiety.

In our attempts to explore the potential mechanism underlying the hepatoprotective effects of these compounds, we assessed the levels of the autophagy-related proteins, Beclin-1 and LC3B, as well as the CYP2E1 enzyme. Autophagy holds a significant role in the context of liver fibrosis, regulated by the activation of autophagy-related proteins, including Beclin-1 and LC3B [10]. Beclin-1 plays a crucial role in autophagy initiation by forming a complex with other proteins [62]. This complex drives the formation of autophagosomes, which are essential for the sequestration of cellular components targeted for degradation [63]. Likewise, LC3B, a pivotal marker of autophagy, undergoes lipidation and is recruited to autophagosomes, facilitating their maturation and cargo degradation [64]. Our findings revealed an upregulation of both Beclin-1 and LC3B in the CCl_4_-treated group, suggesting an elevation in autophagic activity within hepatocytes, which subsequently led to an exacerbated activation of HSCs and the deposition of ECM [9, 65]. Interestingly, our results align with existing studies emphasizing the pro-fibrotic effects of autophagy, through the degradation of lipid droplets within HSCs by lipophagy, a process that supplies the energy essential for the activation and conversion of HSCs into myofibroblasts, ultimately resulting in fibrosis [9, 66, 67]. Upon co-administration of our compounds with CCl_4_, there was a notable reduction in the expression of these autophagy-related proteins. This observation indicates that our compounds play a significant role in inhibiting autophagy, thereby mitigating the development of CCl_4_-induced fibrosis [9]. Conversely, our observed reduction in the expression of Beclin-1 and LC3B following the administration of the compounds under investigation alongside CCl_4_ contradicts some studies proposing the anti-fibrotic effects of autophagy activation [11, 67]. Previous research has suggested that autophagy, when activated, diminishes the accumulation of type I collagen induced by TGF-β activation [68]. However, our results imply that 4-phenyltetrahydroquinolines exert their anti-fibrotic effect by inhibiting autophagy, which is extra active upon CCl_4_ administration [10]. Furthermore, our findings once again demonstrated that compounds **2a** and **2b** exhibit a greater impact on inhibiting autophagy compared to the other compounds tested, effectively suppressing the expression of both Beclin-1 and LC3B.

Regarding CYP2E1, a member of the cytochrome P450 superfamily that participates effectively in the conversion of CCl_4_ into free radicals, silymarin demonstrates a noteworthy decrease in CYP2E1 levels [69]. This reduction in CYP2E1 levels led to a decrease in free radical production and, subsequently, mitigated the induction of liver injury [70]. In contrast, our compounds showed a minimal to negligible impact on diminishing the expression and activity levels of the CYP2E1 enzyme. This implies that 4-phenyltetrahydroquinolines have no effect on the metabolism of CCl_4_ [4].

Our recent findings closely supported our earlier conclusion concerning the significance of various substituents present at the 2-position of the tetrahydroquinoline moiety [18, 71]. It was evident that the presence of an amino group at the 2-position yielded significantly better results compared to the hydroxy substituent [24]. Nevertheless, the limited number of compounds tested was insufficient to validate the conclusion. Accordingly, two more pairs of tetrahydroquinolines, featuring both 2-amino and 2-hydroxy substituents, were synthesized and examined to reassess the previously established conclusion. Fortunately, the outcomes once more demonstrated the superiority of the amino group. Moreover, additional substituents were introduced to the 4-phenyl group, including an amino and a hydroxy group, to further explore whether an amino group at the 4-phenyl ring could compensate the reduced activity observed in the 2-hydroxy compounds. Once more, it became evident that the presence of an amino group at the para-position of the 4-phenyl ring was able to compensate for its absence at the 2-position of the tetrahydroquinoline, with compound **1a** consistently demonstrated superiority over 1b across multiple biochemical tests. Furthermore, the results of the in silico molecular docking of our compounds against Beclin-1, LC3B, and CYP2E1 strongly correlated with the practical testing results, demonstrating enhanced binding of the compound pair containing an amino group at the 2-position of the tetrahydroquinoline moiety.

Finally, while our study provides a promising evidence of the hepatoprotective effects of the synthesized 4-phenyltetrahydroquinoline derivatives in animal models, it is crucial to acknowledge the necessity of clinical trials to bridge the gap between preclinical findings and human application. Clinical trials will provide essential insights into the safety profile, efficacy, and potential therapeutic benefits of these compounds for human liver diseases.

## Conclusion

This study shows that 4-phenyltetrahydroquinolines are beneficial in lowering CCl_4_-induced liver damage and fibrosis in rats. The molecular mechanism behind this hepatoprotective effect is suggested to be through autophagy inhibition, while silymarin achieves this effect by inhibiting both autophagy and CYP2E1 enzyme activity. Furthermore, it was observed that the presence of an amino group in the 2-position of the pyridyl ring (compounds **2a** and **2b**) gave much better results relative to the 2-hydroxyl substituted derivatives (compounds **1a** and **1b**). Such an observation will be further investigated in future studies.

## Data Availability

The data supporting this study’s findings are available from the corresponding author upon reasonable request.
